# Mega-city construction engineering to residential satisfaction: new insights from Yan’ an of China

**DOI:** 10.3389/fpubh.2023.1187519

**Published:** 2023-07-04

**Authors:** Huan Huang, Xin-Meng Qian, Yi Xiao, Zi-Xin Luo

**Affiliations:** ^1^State Key Laboratory of Geohazard Prevention and Geoenvironment Protection, Chengdu University of Technology, Chengdu, China; ^2^Business School, Chengdu University of Technology, Chengdu, Sichuan, China; ^3^College of Management Science, Chengdu University of Technology, Chengdu, Sichuan, China; ^4^School of Journalism and Communication, Chengdu Sport University, Chengdu, Sichuan, China

**Keywords:** residential satisfaction, new district and old district, mountain excavation and city construction, multiple regression analysis, comparative research

## Abstract

**Introduction:**

The contradiction among population, economy and urbanization has gradually intensified, and the Mountain Excavation and City Construction (MECC) project is one of the special solutions. Nevertheless, there are few comparative studies on the project index studies and effect of MECC projects on residential satisfaction. To remedy this deficiency, this study base on the Yan’an new district (YND) reconstruction project, attempting to analyze the specific influencing factors prerelocation and post-relocation from the perspective of residential satisfaction.

**Methods:**

After conducting reliability and validity analysis on each dimension, multiple linear regression and paired t-test were used to analyze and compare the questionnaire data.

**Results:**

The results show that the residential satisfaction index of the YND is indeed higher than that of the Yan’an old district (YOD). Concurrently, the decisive factors of residential satisfaction are also different. Specifically, the interpersonal communication, supporting facilities, community environment and economic income are significant in the YOD, but only the aspect of supporting facilities is negative significant. The supporting facilities, community environment, economic income and urban development are all positive significant in the YND. The satisfaction factors of middle-aged people in YOD and YND have the most significant differences, and the significance of each dimension is different.

**Discussion:**

The research results of this study provide a comparative perspective at the micro-level for evaluating China’s urban construction, and it supplies specific directions for future urban development and the improvement of old cities through the new residential satisfaction index.

## 1. Introduction

The sustainable development goals (SDGs) proposed by the United Nations include many aspects. As a part of SDGs, the urban sustainable development’s main constraints are the human-land conflict, urban construction pollution and urban human settlement environment (1–3). Some studies have explored the issue of urban livability in the context of urban development (4, 5). The study also found that the main characteristics of the human-land conflict are still consistent under different urban backgrounds. For example, both resource intensive cities and highly populated cities reflect the constraints of social and economic development on ecological environment protection (6). The government also adopts various strategies to solve the problems of urban traffic congestion, excessive population density and insufficient resource carrying capacity. In order to deeper understand status of urban research, increasingly researchers include human subjective factors into the scope of investigation, such as residential satisfaction, traffic satisfaction, and happiness (7–10).

The relationship between population and land is always a research hotspot. Although the degree of population growth has not increased significantly, the demand for land use continues to rise (11). Conversely, the carrying capacity of resources continues to decrease. Sustainable land use has become a prerequisite for achieving sustainable development goals in various regions (12). For this situation, many cities alleviate the problems of land use tension through housing planning or urban expansion (13, 14). Researchers are beginning to pay attention to land use efficiency (4, 5). It is worth noting that compared with ordinary urban planning projects, the development of new projects pays more attention to human factors, including interpersonal interaction, daily activities, and place attachment (15, 16). The problem of excessive population density and traffic congestion in Yan’an is also solved by the new district construction plan.

The construction of the Yan’an new district (YND) is to solve the contradiction between population growth and resource utilization. In 2011, the population density of Yan’an reached 59.40% (Statistics Bureau of Yan’an municipal government). To solve the problem of urban land use, Yan’an began to formulate the plan of “Building a city by bulldozing mountains” (Yan’an municipal government) in 2012. There are few studies on this, the largest MECC project in China, and most studies focus on analyzing the characteristics and factors of land settlement caused by the MECC project (17–19). There is little research on the residents’ living conditions in YND from the micro perspective. Moreover, the existing literature on residential satisfaction in the context of urban construction projects mainly focuses on the experience after the completion of the project (20).

Given this deficiency, this study adopts the perspective of residential satisfaction to discuss the impact of this large-scale MECC project (21, 22). The changes brought by urban planning include many aspects. However, through the study of residential satisfaction, the most important human-land conflict could be alleviated (23, 24). Studying the influencing factors of urban residential satisfaction, the key points of improving the rationality, satisfaction and implementation of urban planning could be found. The innovation of this study includes two aspects. Firstly, it studies the impact of the world’s largest MECC project on residents from a micro perspective and measures the quality of project construction by residential satisfaction. Secondly, the comparative study of pre-construction and post-construction in the project is selected to explore the differences in specific influence factors.

The data comes from the questionnaire survey results of the field investigation. The questionnaire is generally divided into two parts: pre-relocation and post-relocation and the design questions are basically the same for comparison. The problem involves residents’ life, education, economy, psychology, urban development and other aspects, with reference to relevant research on satisfaction survey. The question options of both parts are increased from “1″ to “5″. Concurrently, it contains the basic information of the respondents, for instance: age, gender and education.

The structure of this study is as follows. In the following chapters, Section 2 reviews the dimensions of happiness, residential satisfaction, and urban renewal related to satisfaction, with an emphasis on residential satisfaction. It then will present the relevant research on large-scale urban projects. On this basis, the analysis framework of residential satisfaction is introduced. In Section 3, the data sources and methods are drawn into. Section 4 presents the results of descriptive analysis, t-test, and multiple linear regression analysis. Subsequently, the research results are discussed in Section 5. In the last section, the study summarizes the academic and policy implications of the study.

## 2. Literature review

Satisfaction is studied as an intermediary variable affecting life and well-being (25, 26). For instance, the study found that the closer the residence is to the green space, the higher the living satisfaction and the stronger their life happiness. On the contrary, the farther the house is from the green space, the lower the results of residential satisfaction and life happiness (27). As a sub-dimension of satisfaction evaluation, residential satisfaction index reflects people’s satisfaction with the living environment. Researching the influencing factors of residential satisfaction may improve people’s future living conditions (28).

Residential satisfaction has been widely proved to be affected by the characteristics of residential or community environments (29–31). It has been demonstrated that the more frequent the activities of the neighborhood or family, the people’s residential satisfaction may be higher (32). In addition, the number of parks beside the residence also affects the satisfaction of the residences, and superior facilities in parks could bring about a greater impact on satisfaction (33). Nevertheless, the residential satisfaction in diverse backgrounds is still different. Research has involved many backgrounds such as suburb and traditional environment, public low-rent housing and urban planning impact (34–36). In particular, with the development of urbanization in China, it is more likely that those with housing ownership have higher living satisfaction than tenants (37).

A large and growing body of literature has investigated the impact of various urban development planning policies on residential satisfaction (8, 38, 39). Some researchers are dedicated to large-scale MECC projects, and discusses residents’ lives and urban development from the perspective of residential satisfaction (40, 41). In developing countries, residents’ satisfaction is affected by the government planning system, although with varying degrees (35, 42). Furthermore, the population flow and the changes in housing environment promoted by urban planning have significantly changed residential satisfaction (43, 44). It is worth noting that the residential satisfaction after reconstruction or land expansion may decline, although the living environment and urban development have improved (15, 16). Existing studies have also found that in the urban expansion stage, the reconstruction process and results of these communities also have an essential impact on the assessment of residential satisfaction, and urban development is also considered to be an important factor affecting satisfaction (15, 16).

Part of the research focuses on urban renewal and residential satisfaction. It is clearly proposed that urban development and renewal will affect residents’ housing satisfaction (45–48). Research has found that urban renewal brings about changes in social capital, which is positively correlated with changes in residential satisfaction (46). In addition, some scholars have focused their research on older adults and found that their living satisfaction is positively affected by the living environment during urban renewal (48). The different modes of urban renewal can also affect residential satisfaction, and affect satisfaction through factors such as residential conditions, neighborhood relationships, and economic expectations (49). For the sponge city renewal caused by floods, it was found that the performance of young and older adult populations in terms of housing satisfaction is different (47).

**Figure 1:**
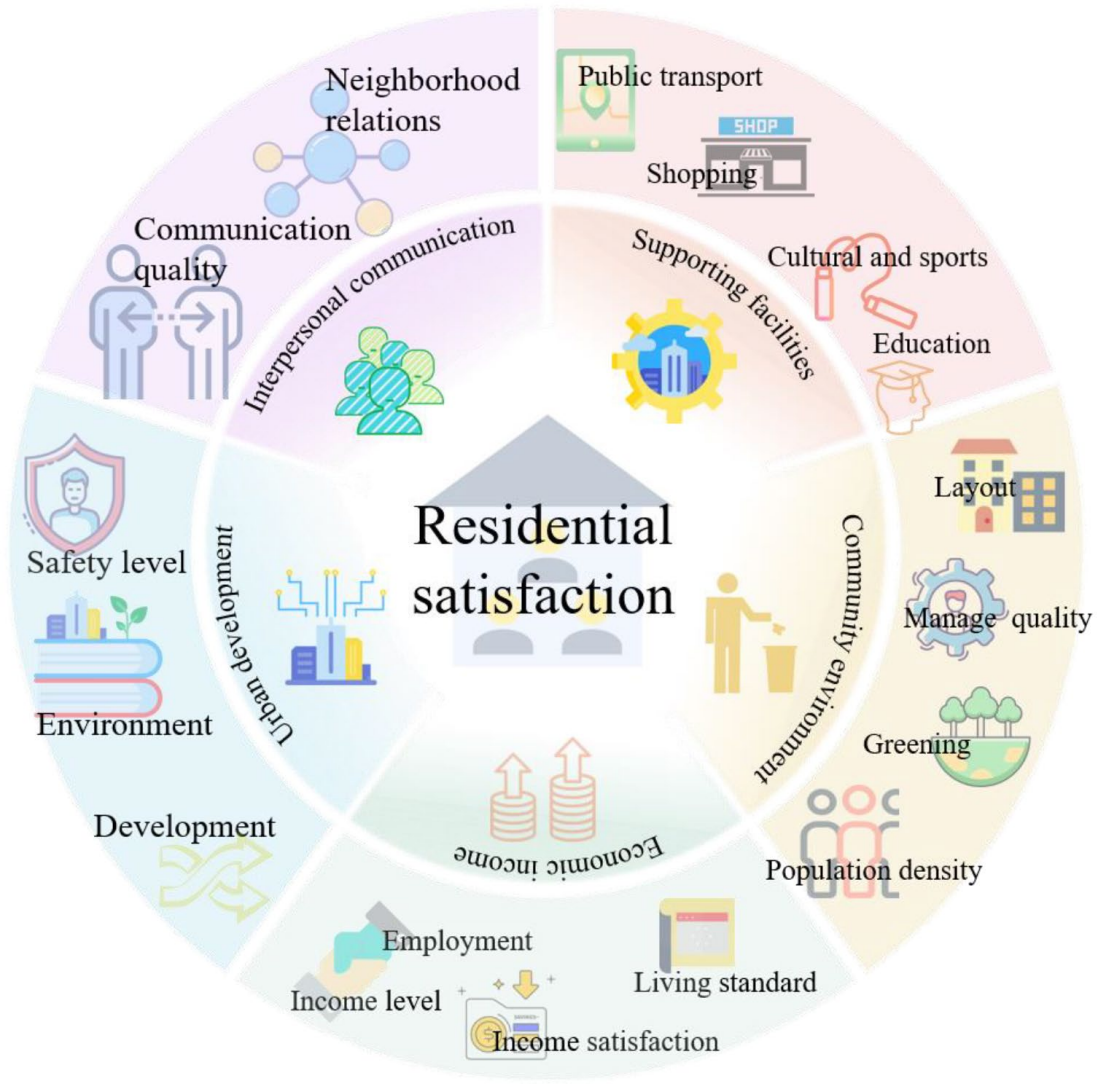
Analysis framework of residential satisfaction.

As there is uncertainty in the measurement of large-scale MECC projects in cities, this study chooses the residential satisfaction index to analyze the impact of the MECC project on actual life (50). In addition, the perceived change of satisfaction is the feedback on the effectiveness of urban planning projects (51). Figure 1 shows the dimensions of theoretical analysis of residential satisfaction. The residential satisfaction is structured from multiple dimensions, and each dimension is not only determined by a single indicator. Previous studies have shown that the determinants of residential satisfaction generally consider three aspects: sociodemographic variables, residential environment characteristics, and housing characteristics (38, 43, 52). Concurrently, it has been demonstrated that the changes in interpersonal communication brought by the residential environment’s alterations also have an impact on residential satisfaction (31, 44). Additionally, some scholars have studied issues related to social capital factors and residential satisfaction in consideration of urban renewal (53). Therefore, in addition to the above related factors, economic and urban development factors are also included in the satisfaction evaluation (54).

Ultimately, the research on residential satisfaction has involved various fields and aspects. In particular, this study focuses on summarizing the relevant research on residential satisfaction in the context of urban development. The current research mainly concentrates on the residential satisfaction evaluation after the completion of the planning project and the intermediary effect of other factors on residential satisfaction (8, 38, 41). Nevertheless, there are still few comparative studies on residential satisfaction for pre-changes and post-changes in the residential environment. Therefore, in order to deeper understand the impact of such large-scale urban engineering projects, the specific discrepancies between the pre-project and the post-project are compared. Simultaneously, this study conducts a field survey on the MECC project in Yan’an, and deconstructs the actual impact from the perspective of residents’ satisfaction to evaluate the project effect.

## 3. Materials and methods

### 3.1. Study area

**Figure 2:**
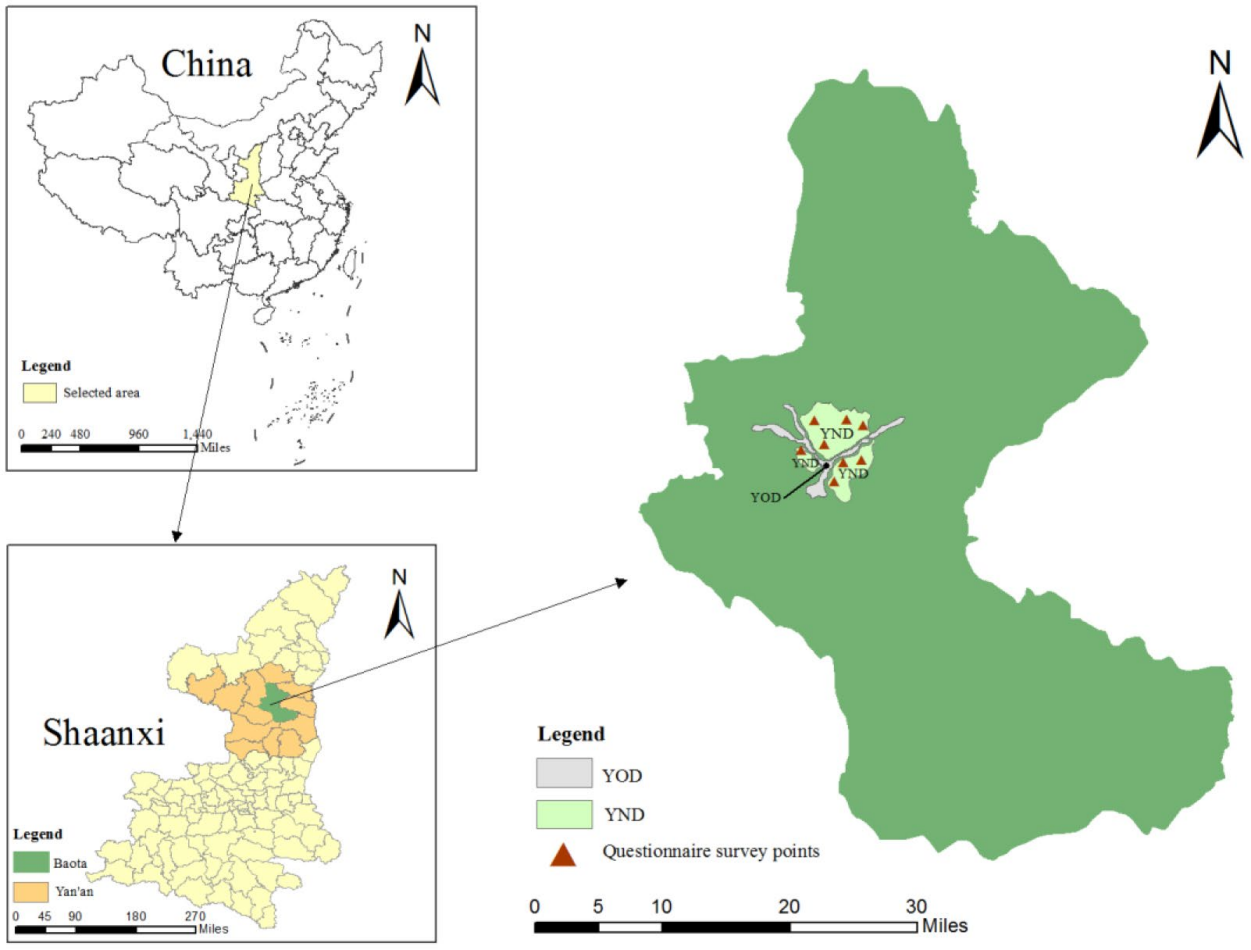
The regional location of the YND and YOD.

Yan’an is located in the north of Shaanxi Province, and its urban center is placed in the hilly and gully region of the Loess Plateau. Due to the particular geographical location of the three mountains nearby, the urban district of Yan’an presents a “Y” shaped construction. The most spacious road in Dongchuan can reach 20 kilometers, but the widest place is less than 1 km. Before the construction of YND, there were nearly 500,000 people placed in such a narrow urban district. Despite being an underdeveloped city in Western China, the population density of Yan’an is close to Beijing and Shanghai. Facing the problems of crowded population, urban development, and limited land use, Yan’an passed the planning project of “Building a new city on the mountain,” that is, flattening the surrounding mountains, filling the ravines between the mountains, and building a new urban district to redevelop the district of the old district, as shown in Figure 2–a regional comparison of the YND and YOD. As the most extensive geotechnical engineering in the collapsible Loess region in Asia and even the world, the impact of the YOD redevelopment on residents is worthy of in-depth study.

### 3.2. Data collection

The premise of the study is that the residents with living experience of the YND and YOD pre-residential satisfaction and post-residential satisfaction can be evaluated and compared. Therefore, the questionnaire survey is only conducted for residents who have moved into the YND from YOD and have lived for a certain period. The field survey took place in July 2021, 8 years since the completion of phase I construction of YND (2013) and 3 years since the overall relocation of 160,000 residents in Yan’an (2019). The investigators distributed the questionnaire within the scope of YND, furthermore in an attempt to increase the validity of the questionnaire results by asking “whether they are an original resident?” a brief one-to-one explanation is given in different residential communities. The respondents can participate in this survey by oral answers, actively filling in the study questionnaire, or scanning the code to obtain an electronic questionnaire. A total of 266 completed questionnaires (see Table 1) were collected in the initial stage, and a small number belonging to non-local residents were excluded from the analysis. Furthermore, considering the analysis process, a multi-dimensional mean t-test is carried out. If the mean value is used to replace the blank value of some questionnaires, it will have a significant impact on the results and for this reason all of the blank questionnaires were excluded. The final number of valid questionnaires was 210 (see Table 1).

**Table 1:** Respondents’ numerical demographic characteristics.

	Mean	Median	S.D.	All questionnaires	Non-local	Blank	Valid
				Unit/copies			
Age	32.49	30	11.604	266	30	56	210
Family population	3.83	4	1.322				

#### 3.2.1. Interpersonal communication

Urban planning will not only directly change the living environment but also have an impact on interpersonal relations, especially the establishment of new cities. The satisfaction of daily interactions will improve residential satisfaction. Table 2 ( $X_{1}$ ) is a questionnaire for interpersonal communication.

**Table 2:** Evaluation indicators for the sub-dimension of residential satisfaction.

Dimension	Subindex	Questions	Scores
Interpersonal communication (X_1_)	Neighborhood relations (X_11_)	How harmonious are your neighbors?	(1–5)
	Communication quality (X_12_)	How satisfied are you with your interpersonal relationship?	(1–5)
Supporting facilities (X_2_)	Public transport (X_21_)	How satisfied are you with the distribution of public transport?	(1–5)
	Cultural and sports (X_22_)	The rationality of the layout of cultural and sports facilities.	(1–5)
	Education (X_23_)	The rationality of distribution of educational resources.	(1–5)
	Shopping (X_24_)	How satisfied are you with the supporting facilities of commercial supermarkets?	(1–5)
Community environment (X_3_)	Population density (X_31_)	How satisfied are you with the population density of the community?	(1–5)
	Greening (X_32_)	How satisfied are you with the green area of the community?	(1–5)
	Management quality (X_33_)	How satisfied are you with the service quality of the residential property?	(1–5)
	Layout (X_34_)	How satisfied are you with the arrangement of building spacing in the community?	(1–5)
Economic income (X_4_)	Income level (X_41_)	Overall income level.	(1–5)
	Employment (X_42_)	How satisfied are you with the job opportunities?	(1–5)
	Income satisfaction (X_43_)	How satisfied are you with your income?	(1–5)
	Living standard (X_44_)	How satisfied are you with your living standard?	(1–5)
Urban development (X_5_)	Safety level (X_51_)	How satisfied are you with the urban security situation?	(1–5)
	Environment (X_52_)	How satisfied are you with the urban environment?	(1–5)
	Development (X_53_)	How satisfied are you with the comprehensive development of the city?	(1–5)

#### 3.2.2. Supporting facilities

Improving the conditions of supporting facilities would improve the residents’ living satisfaction. Life mainly includes shopping, education, housing, and transportation. Therefore, the specific design items of the questionnaire for supporting facilities are shown in the following Table 2 ( $X_{2}$ ).

#### 3.2.3. Community environment

Both subjective and objective evaluations of the living environment will have an impact on satisfaction (55). Especially in the new urban construction, planning the community environment in advance could improve living satisfaction. Table 2 ( $X_{3}$ ) is a questionnaire for the community environment.

#### 3.2.4. Economic income

Both new and old districts can improve the quality of life and subjective satisfaction by improving their income level. The questionnaire questions about economic income are shown in Table 2 ( $X_{4}$ ).

#### 3.2.5. Urban development

Suppose the residents’ expectations are taken into account in advance in the construction or planning of new cities and are satisfied as much as possible. In that case, the psychological gap of the residents could be reduced, and improving satisfaction. The specific questions of the questionnaire for urban development are shown in Table 2 ( $X_{5}$ ).

#### 3.2.6. Measurement of residential satisfaction

The measurement of both residential satisfaction and influencing factors is equally essential (30). Instead of using only one question to determine residence satisfaction, it would be preferable to choose multi-point questions to determine satisfaction (52). Considering the previous research results, it is believed that matching the building environment with daily activities would promote community residential satisfaction (56). Therefore, the actual residential satisfaction after moving into YND is inquired by three aspects related to the environment: area, house type, and noise. Accordingly, four questions are used to jointly determine the residential satisfaction ( Y ) of YND (see Table 3).

**Table 3:** Specific questions of residential satisfaction.

Questions	Scores
1. How satisfied are you with the housing type?	(1–5)
2. How satisfied are you with the living area?	(1–5)
3. How satisfied are you with the residential noise?	(1–5)
4. How satisfied are you with your residence?	(1–5)

The mean value of the four questions ultimately represent the residential satisfaction of YND and YOD. All survey questions utilize a 5-point Likert scale, and the values of the degree increased from “very dissatisfied” to “very satisfied.” It is generally believed that an alpha value higher than 0.7 can judge that the index conforms to one center and has a strong correlation (57). These four questions have passed the reliability uniformity test of the questionnaire (Cronbach’s alpha = 0.852). The reliability results of other questionnaires are shown in Table 4.

**Table 4:** Reliability test results of five dimensions.

Dimensions	Cronbach’s alpha	
	YOD	YND
Interpersonal communication ( $X_{1}$ )	0.731	0.729
Supporting facilities ( $X_{2}$ )	0.809	0.786
Community environment ( $X_{3}$ )	0.754	0.803
Economic income ( $X_{4}$ )	0.834	0.796
Urban development ( $X_{5}$ )	0.728	0.811

### 3.3. Multiple linear regression method

#### 3.3.1. Index classification

The data collected in 3.2 are divided into five categories: interpersonal communication, supporting facilities, community environment, economic income, and urban development. Analyzing the impact of various factors on residential satisfaction ( Y ). Each aspect contains multiple sub-dimension indicators, and the mean value of the sub-indicators represents the value of each dimension. Specifically, interpersonal communication ( $X_{1}$ ) only contains two sub-indicators, because it is found in the reliability test that Cronbach’s alpha is 0.731 after deleting the secondary indicator “number of meals,” which is much larger than the three sub-dimensions (Cronbach’s alpha = 0.505); Supporting facilities ( $X_{2}$ ) have four sub-indicators; the community environment ( $X_{3}$ ) is measured by four secondary indicators; economic income ( $X_{4}$ ) also has four sub-indicators; urban development ( $X_{5}$ ) is determined by three secondary indicators. The specific Cronbach’s alpha values of each dimension are shown in Table 4.

#### 3.3.2. Multiple linear regression

Linear regression analysis is a common method for studying the relationship between dependent and independent variables. When the number of independent variables is only one, it is a simple linear regression analysis, entitled univariate linear regression analysis. Its model expression is as follows:

(1) $$Y=\beta_{0}+\beta_{1}X+\varepsilon$$

In equation (1), Y is the dependent variable and X is the independent variable. The intercept of the line on the Y -axis is $\beta_{0}$ , $\beta_{1}$ represents the regression coefficient of X . The larger the absolute value of the coefficient, the greater the influence of the independent variable on the dependent variable. The ε express the error of the equation. However, multiple linear regression is adopted if there are two or more independent variables. Equation (2) shows a multivariate linear regression model.

(2) $$Y=\beta_{0}+\beta_{1}X_{1}+\ldots \beta_{n}X_{n}+\varepsilon$$

Where $X_{i}\left(i=1,2,\cdots ,n\right)$ represents multiple independent variables and Y represents the unique dependent variable. $\beta_{i}$ represents the partial regression coefficient, and means the influence of the change of the $i_{th}$ variable ( $X_{i}$ ) on the dependent variable Y when other independent variables remain unchanged. When there are n groups of observed samples, it very commendably may be expressed in matrix form, as shown in equation (3), $Y_{i}\left(i=1,2,\cdots ,n\right)$ represents the dependent variable in each group.

(3) $$\left[\begin{array}{c} Y_{1}\\ Y_{2}\\ \vdots \\ Y_{n} \end{array}\right]=\left[\begin{array}{cccc} 1 & X_{21} & \cdots & X_{k1}\\ 1 & X_{22} & \cdots & X_{k2}\\ \vdots & \vdots & \cdots & \vdots \\ 1 & X_{2n} & \cdots & X_{kn} \end{array}\right]\left[\begin{array}{c} \beta_{1}\\ \beta_{2}\\ \vdots \\ \beta_{k} \end{array}\right]+\left[\begin{array}{c} \varepsilon_{1}\\ \varepsilon_{2}\\ \vdots \\ \varepsilon_{n} \end{array}\right]$$

The n is much greater than k . Statistical analysis is performed using SPSS software (version 26). In the model construction of the regression equation, Y represents residential satisfaction, $X_{i}\left(i=1,2,3,4,5\right)$ represents interpersonal communication, supporting facilities, community environment, economic income, and urban development. Besides, a t-test is used to test the regression coefficient to judge the significance and visualized it with Graph Pad software (version 8).

## 4. Empirical results

### 4.1. Descriptive statistical results

In addition to the numerical demographic variables (see Table 1), the classified demographic statistical results of the respondents in the field survey are set out in Table 5. The questionnaire process is random and one-on-one to ensure the authenticity and integrity of the data. Therefore, the overall situation of residents in YND could be estimated from the condition of the questionnaire object. As shown in Table 5, the respondents who belong to urban districts is higher compared to to rural districts, which could reflect that most population of YND may belong to urban districts. The overall level of education expresses excellent, and the respondents with higher education account for more than 60%. From these data, it could be estimated that most residents belong to the masses. What protrudes in Table 5 is the overall health of residents in YND is benign. Married and unmarried groups account for half of the total respondents. The employment situation of residents in YND reflects diversity.

**Table 5:** Categorical demographic variables of respondents.

Variable	Category	Frequency	Percentage
Account type	City	134	63.8%
	Countryside	76	36.2%
Education level	Primary school	5	2.4%
	Junior high school	28	13.3%
	High school	39	18.6%
	Bachelor, junior college or above	138	65.7%
Political outlook	Masses	153	72.9%
	Party member	55	26.2%
	Democratic parties	2	1%
Health condition	Commonly	37	17.6%
	Quite good	75	35.7%
	Excellent	98	46.7%
Marital status	Unmarried	94	44.8%
	Married	111	52.9%
	Divorce	4	1.9%
	Other	1	0.5%
Occupation	Party and government organs	23	11%
	Public institution	40	19%
	Enterprise	42	20%
	Freelance	45	21.4%
	Retiree	4	1.9%
	Student	35	16.7%
	Other	20	9.5%

### 4.2. *T*-test analysis results

#### 4.2.1. *T*-test results of residential satisfaction

**Figure 3:**
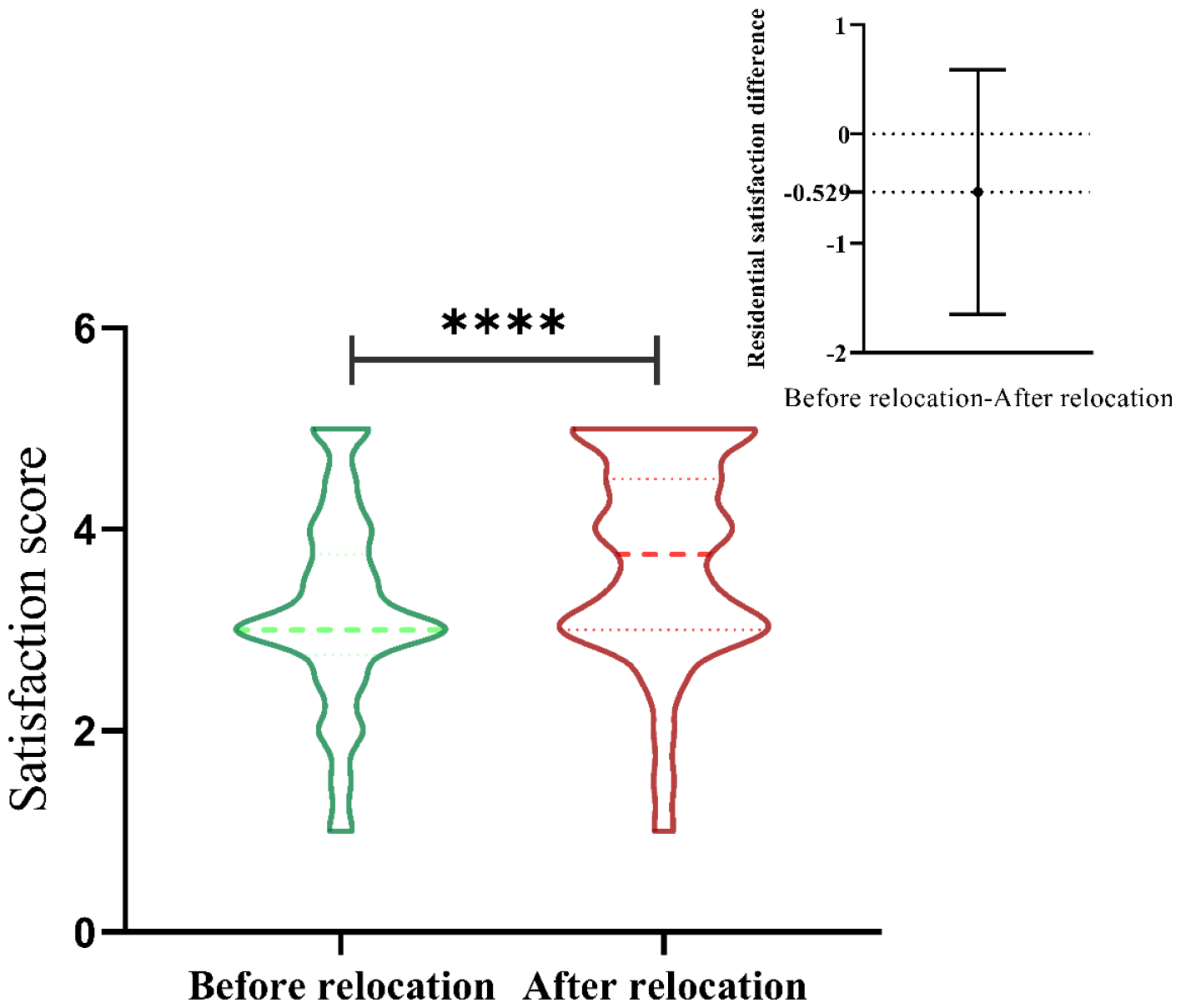
The results of the difference test (*t*-test) of residents’ living satisfaction between the old city and the new city in Yan’an. Living satisfaction score is the mean value of the questions shown in; The thumbnail in the upper right corner presents the difference by subtracting the score of the new district from the score of the old district; ****means *p* < 0.0001.

The violin chart reflects the overall distribution of residents’ satisfaction scores when living in old and new districts. It is apparent from Figure 3 that there is a significant difference in the residential satisfaction of living in the YOD and YND. The figure in the upper right corner presents a thumbnail of the difference between the scores of the old-new districts. The average satisfaction of the YND is higher than that of YOD, although the scoring mode is basically the same. Furthermore, the scores of the YND are mostly 3 points or above, while the scores of YOD are mainly 3 points, especially in the gap of 5 points.

#### 4.2.2. *T*-test results of five sub-dimension

**Figure 4:**
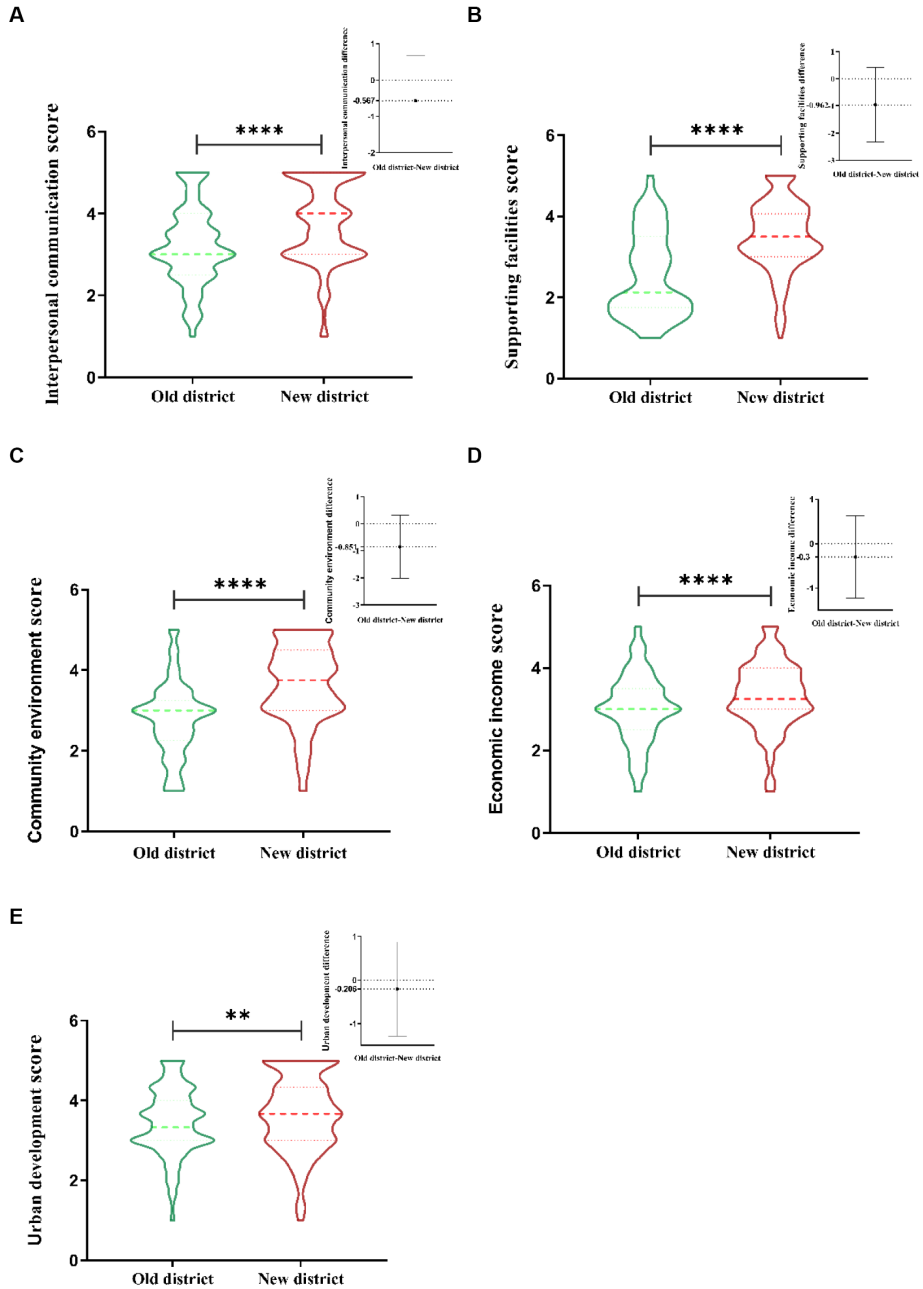
Comparison chart of five sub-dimension data. The score adopts the mean value of each sub-dimension evaluation item; The thumbnails in the upper right corner show the difference by subtracting the score of the new district from the score of the old district. ** means *p* < 0.05; **** means *p* < 0.001.

The score difference results of the five sub-dimensions affecting satisfaction are shown in Figures 4A–E, including interpersonal communication, supporting facilities, community environment, economic income and urban development. The thumbnails of the score difference results are shown in the upper right corner of the figure. Despite the score difference of urban development being slightly small, (*p* < 0.05), the results of other dimensions were significantly different (*p* < 0.0001). From the perspective of visualization, the five sub-dimensions show different outcomes. In the interpersonal dimension (see Figure 4A), the score of the new district presents a “dumbbell shape,” with large ends and small middle. The data of old district decreases from the middle to both ends, showing a “wavy” reduction. In the dimension of supporting facilities (see Figure 4B), the data of new district is represented by “inverted vase,” and that of old district is expressed by “positive vase.” Moreover, the difference value of the old-new districts is the largest among the five dimensions, which is 0.962. In the community environment dimension (see Figure 4C), the obvious difference between the new and old districts is that the scores of YND are more concentrated at more than 3 points, and the distribution is relatively uniform. Most of the scores in the old district are below 3 and mainly distributed at 3 points. The contrast between new and old districts in the visualization results of economic income (see Figure 4D) is relatively minimal, and the figure gradually decreases from the middle to the both ends. Although the smallest difference in the five dimensions is urban development, with a difference of 0.206, the distribution difference of the scores (see Figure 4E) is still obvious. Specifically, the proportion of high scores in YND is more conspicuous.

### 4.3. Regression analysis results

The specific impacts of the five sub-dimensions on residential satisfaction are shown in Table 6 below. By considering the differences in satisfaction perception among different age groups, the study population is divided into young people (Years ≤44), middle-aged people (45 ≤ Years ≤59), and older adults (Years ≥60) based on international age classification standards. It is to be regretted that due to insufficient statistics on questionnaires over the age of 60, the focus of the discussion is on analyzing the overall satisfaction results and the perceived differences between the young and middle-aged populations. The results show that the influence of only four dimensions is significant in both the old and new districts, although the significant dimensions are different. Specifically, the residential satisfaction of the YOD is positively correlated with interpersonal communication, community environment and economic income but negatively correlated with supporting facilities. The performance of the urban development dimension has no significant impact. The residential satisfaction of YND is positively correlated with the supporting community, community environment, economic income and urban development, while the interpersonal dimension has changed from significant to insignificant. The living satisfaction of young and middle-aged people in YOD is significantly influenced by interpersonal communication, supporting facilities and urban development dimensions, while their living satisfaction in YND is only significantly affected by economic income. In terms of community environment and economic income, there is a significant difference in the impact of living satisfaction between young and middle-aged people in the YND and YOD. The community environment only affects the living satisfaction of middle-aged people in the YND, while economic income only affects the living satisfaction of young people in YOD. It should be noted that demographic variables is excluded from the regression analysis, although demographic variables is considered in the data collection. Because the initial experimental results shows that such variables had no significant relationship with residential satisfaction, especially in the comparative analysis of new and old districts. Therefore, the discussion part only analyzes the changes in residential satisfaction in new and old districts from the impact of five sub-dimensions.

**Table 6:** Results of sub-dimensional regression analysis of residential satisfaction considering the influence of age.

	Age group	Interpersonal communication	Supporting facilities	Community environment	Economic income	Urban development
		B	B	B	B	B
Old district	Years ≤44	−0.706^***^	1.487^***^	0.032	0.182^***^	−0.492^***^
	45 ≤ Years ≤59	−0.532^****^	1.728^****^	0.016	0.055	−0.612^****^
	Years ≥60	−1.095	1.875	0.004	−0.224	−0.246
Total		0.335^****^	−0.071^***^	0.128^***^	0.600^****^	0.041
New district	Years ≤44	0.074	0.088	0.028	0.588^****^	0.047
	45 ≤ Years ≤59	0.117	0.242	0.624^***^	−0.344^*^	0.062
	Years ≥60	−0.163	0.594	−1.164	−0.032	1.374
Total		−0.033	0.465^****^	0.038^**^	0.415^****^	0.234^****^

Dependent variable: residential satisfaction, B is the regression coefficient. *p < 0.1; **p < 0.05; ***p < 0.01; ****p < 0.001.

**Figure 5:**
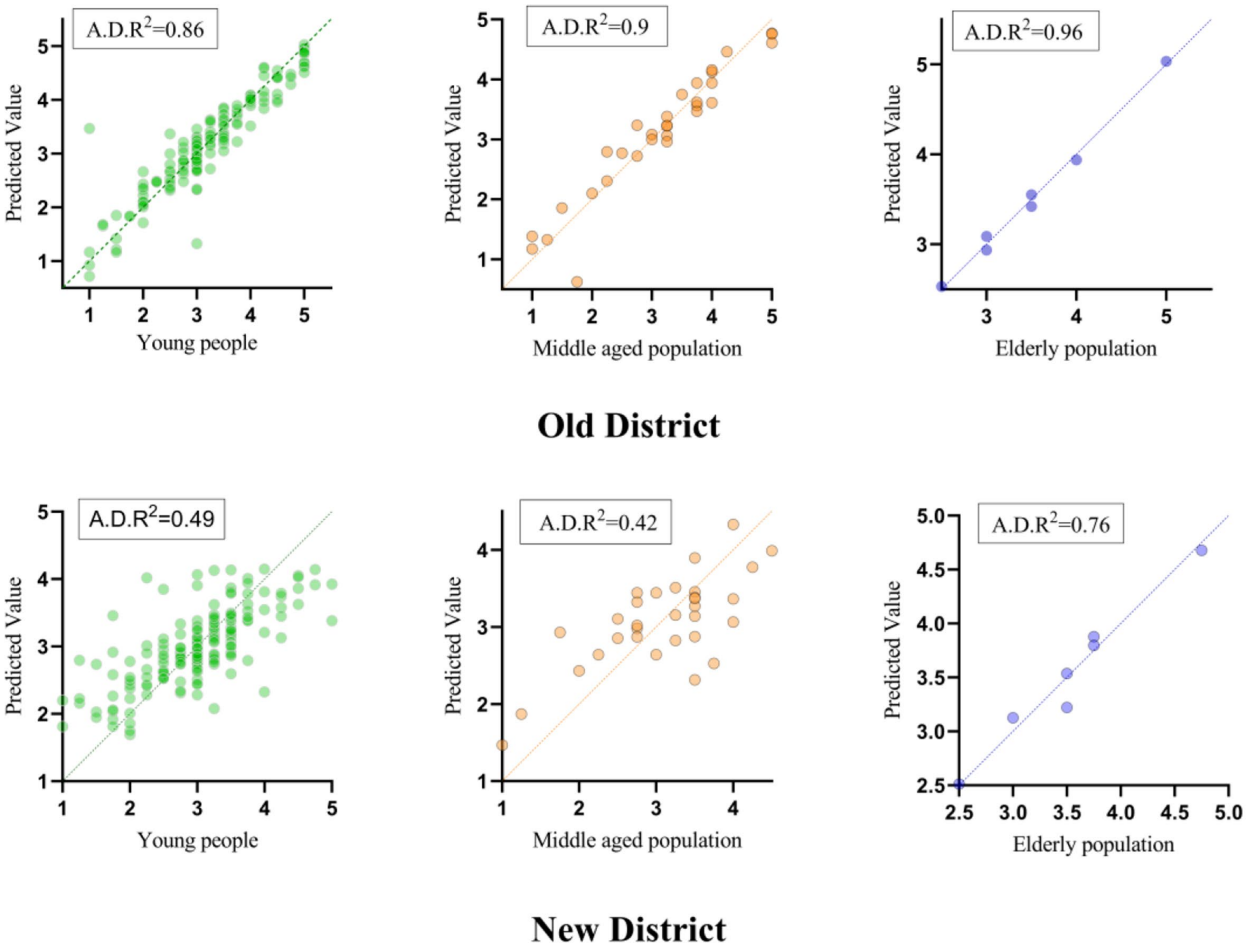
Multiple linear regression diagram of the new district and old district by age groups. A.D. *R*^2^ means Adjusted R square.

Simultaneously, the visual results of multiple regression analysis (see Figure 5) show the predictive ability and the actual results of residential satisfaction among different age groups in the YND and YOD. The value of YND is more obvious in the high segment (3–5) compared with the regression map of YOD. According to the results of the standardization coefficient (B), the most significant impact on the residential satisfaction of the old district is “economic income (0.6),” followed by “interpersonal communication (0.335)” and “community environment (0.128).” Although the impact of “supporting facilities” on satisfaction is negative, the coefficient value is the smallest (−0071). The standardization coefficients of all dimensions of the YND are positive (see Table 6). The “supporting facilities (0.465)” has the greatest impact on residential satisfaction, and the order of the remaining dimensions is “economic income (0.415)” > “urban development (0.234)” > “community environment (0.085).” According to the standardized coefficient results (B) under the age group dimension, the residential satisfaction of young people in the YND is significantly more affected by economic income (0.588) than in YOD (0.182). The significance of various dimensions varies for middle-aged people in new and old districts. The findings for older adults was not analyzed.

**Figure 6:**
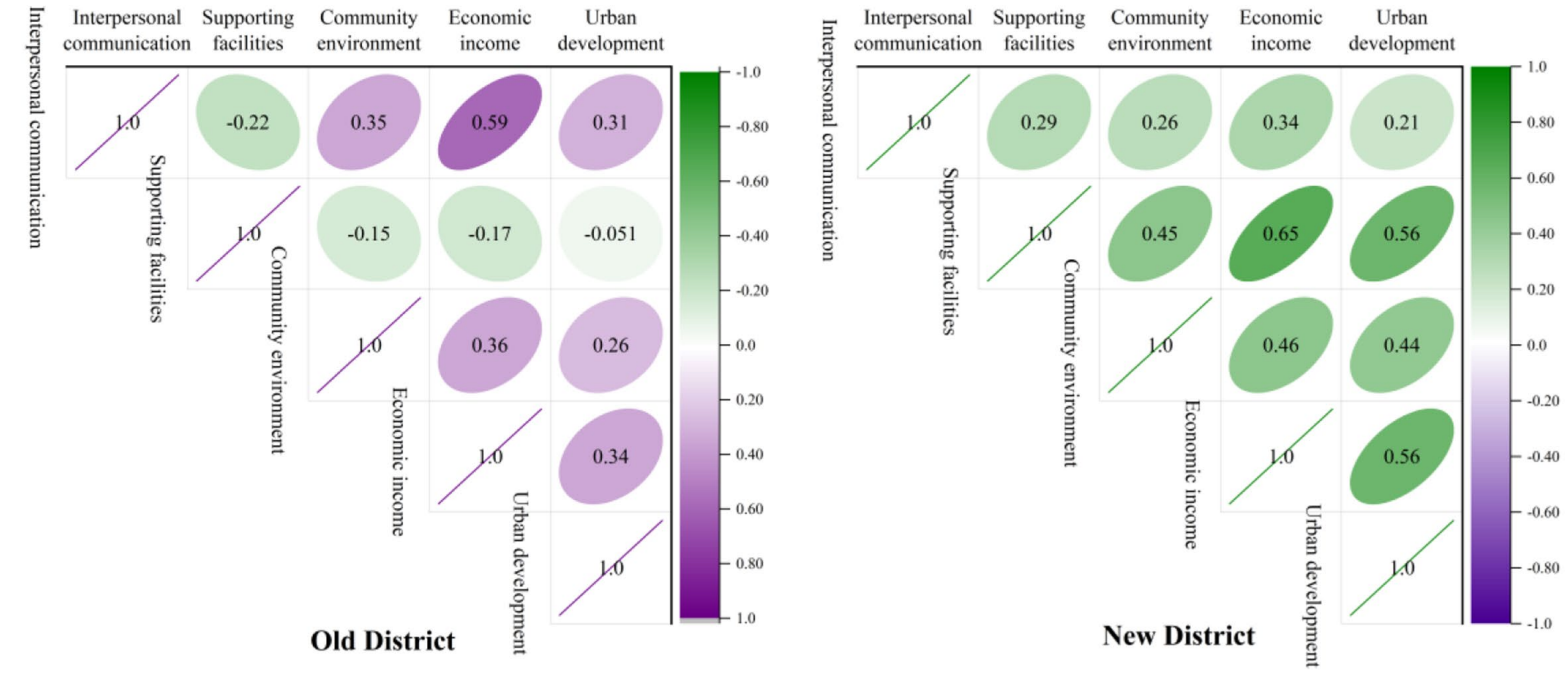
Heat map of correlation between factors. Numerical values represent the magnitude of the correlation, “-” and “+” values represent the effect of the influence (The “+” sign is not displayed).

Simultaneously, the validity of regression analysis and multi-collinearity are considered. The article adopts inter-factor validity analysis for validation and is reflected through the heat map below (Figure 6). On the five dimensions of data in the new and old districts, the biggest correlation is between the economic income and supporting facilities of the new district (0.65). However, referring to previous studies, it is believed that a correlation coefficient below 0.7 can exclude multiple collinear effects between factors, so it can be included in the study. The correlation of all other dimensions is less than 0.6, and most of the absolute values are around 0.3 meaning that the data value is worth studying.

## 5. Discussion

This section discusses the differences in residential satisfaction in the YOD and YND from the perspective of five sub-dimensions in detail. The impact of supporting facilities on residential satisfaction shows significant differences in both YOD and YND. In fact, a strong relationship between supporting facilities and residential satisfaction has been reported in the literature (58). The results of multiple regression analysis show that the preferable supporting facilities in YND may produce a higher result in residential satisfaction. One unanticipated finding is that the improved supporting facilities in YOD would reduce satisfaction, which is inconsistent with the research in YND (35). Although the equipment in the old district generally does not undergo comprehensive renewal. This inconsistency could be attributed to community facilities which gather people to produce noise and commotion. In addition, YOD may defective sound insulation. Moreover, noise and commotion not necessarily caused by the construction process of the facility but rather by the living noise generated by people’s leisure gatherings after the facility is completed. Moreover, the community facilities of the YND have gradually improved over time, therefore people are more inclined to live in new communities with more supporting facilities and preferable conditions.

Consistent with some research results, the community environment can significantly affect residence satisfaction (59, 60). The results of this study show that excellent community environment would positively promote residential satisfaction, whether in the YOD or YND. The significance is stronger in YOD (*p* < 0.0001). In terms of the influence degree, it has a greater impact on the residential satisfaction of YOD (*B* = 0.128) and a smaller impact on YND (*B* = 0.085). This may indicate that there is little difference between the environment of each community in the YND, and it is jointly planned and designed by the government. Therefore, the residents of YND do not have a strong sense of environmental differences, so the regression coefficient is relatively small.

The same significance is also reflected in the dimension of economic income, which shows a significant positive correlation with residential satisfaction (*p* < 0.0001). This result corroborates the findings of a great deal of the previous research results [Chen et al., 2013; (38)]. Specifically, among the five dimensions, economic income has the greatest impact on the residential satisfaction of YOD (*B* = 0.6), and the impact of economic income on YOD (*B* = 0.6) is greater than that of YND (*B* = 0.415). The reason may be that in YOD, residents have lower expectations of changes in other dimensions (*B* < 0.4), making it difficult to make significant changes, thus highlighting the impact of economic income due to the smaller impact. The desire for economic income will drive residential change performance (61, 62). In YND, the changes in various dimensions of life are more significant than before, and the impact of a single economy is relatively weak. It seems possible that this significant impact may be due to YOD having older living hardware facilities and a long construction history. The community construction of the planned YND is not different compared with YOD, with the result that people would pay more attention to their own economic situation. Therefore, more attention should be paid to improving the economic income for residents, including economic sources and employment opportunities (63).

In the dimension of interpersonal communication, the significant impact on residential satisfaction is only for YOD. This difference may be caused by some changes in the social interaction between residents after moving into YND, which in some cases completely changes (15). Unfamiliar environments and people will have an impact on residential satisfaction (31, 44). The results of the study found that more harmonious interpersonal communication would positively promote residential satisfaction in YOD, which confirms the previous research (53). Although the residential satisfaction of the YND is not significantly affected by interpersonal communication, these results also support evidence from previous urban research results (59). A possible explanation for these results could be attributed to the neighborhood relations and communication quality of YND which does not yet match the actual situation of YOD.

The same significant difference is also reflected in the dimension of urban development. The current study found that only the residential satisfaction of YND is positively and significantly affected by urban development. The impact of urban development on residential satisfaction in YOD is not significant. This inconsistency may be related to less investment in public security management and community environmental management in YOD (64, 65). Another possible explanation for this is that the residents who move to YND have further expectations for future urban development, and the overall “development satisfaction” in YND is higher than that of YOD. The t-test results show that the residents of YND have significantly higher scores on the “urban development” of the new district than in the old district. In the sense of development, residential satisfaction could be improved by studying urban development, such as improving community security and comprehensive development under the background of no major construction projects in cities.

On the one hand, the most obvious finding to emerge from this study is that there is a significant difference in the level of residential satisfaction in YOD and YND. The residential satisfaction of YND is significantly higher than that of YOD. In other words, this result confirms that urban reconstruction plays an obvious role in improving the residential satisfaction. Based on the perspective of urban development, the impact of large-scale engineering construction on residential satisfaction is clarified in this study. On the other hand, the comparison results of the satisfaction’s influencing factors between the YND and YOD express how each factor affects the residential satisfaction. What is more important, these influencing factors, such as community spacing and employment opportunities, could be taken into account in the future re-planning or construction of cities.

## 6. Conclusion

Based on the multiple linear regression model and the analysis framework of residential satisfaction, this study takes Yan’an as a case that has implemented the mega urban construction project (MECC). The 210 effective questionnaires collected are studied quantitatively. By analyzing and clarifying the relationship between the influencing factors of each dimension of the residential satisfaction, it is hopes to improve the matching degree between urban construction and residents’ expectations. It is expected to provide theoretical and practical significance for future urban planning and construction, especially for cities planned to be rebuilt and updated.

The survey results show that the overall residential satisfaction after moving into YND is higher than that in YOD. In addition, this study found that the impact of each dimension in the pre-relocation and post-relocation for residential satisfaction is also different, which indicates that the residents of Yan’an have unique expectations for the YND. The findings of this study have many practical implications. From one viewpoint, the exploration of this study would assist governments with clarifying the dimension of the real impact on residents’ actual living, and create a more livable, coordinated and sustainable living environment to improve residential satisfaction. It likewise gives explicit measures for the development of ordinary communities. This study contributes to governments understanding of the needs of the residents, and might enable the inhabitants to obtain a true sense of participation, satisfaction, happiness and security in the future. For example, the future planning of the new town could pay attention to the supporting facilities of the community and the living experience, such as culture and supermarkets. For another, although the facilities in the old city are defective, satisfaction with living could be improved by improving community management and increasing investment in local economic enterprises. Meanwhile, considering the differences in satisfaction among different age groups, urban development could consider the expectations of young people for future economic income, as well as the environmental requirements of middle-aged people. Although existing studies have considered the application of residential satisfaction as a key factor in urban planning, it lacks application in Yan’an MECC project. This study could provide a reference for the actual implementation of future new district planning from the perspective of residential satisfaction.

## Data availability statement

The data analyzed in this study is subject to the following licenses/restrictions: Request from corresponding author on reasonable request. Requests to access these datasets should be directed to huan77@126.com.

## Author contributions

HH: funding acquisition, investigation, project administration, and resources. X-MQ and YX: conceptualization, data curation, methodology, visualization, roles writing–original draft, and writing–review and editing. Z-XL: validation, supervision, and investigation. All authors contributed to the article and approved the submitted version.

## Funding

This work was supported by the National Natural Science Foundation of China (41790445); Sichuan Province Philosophy and Social Science Planning Major Project, China (SC22ZDYC45); Open fund of State Key Laboratory of Geohazard Prevention & Geoenvironment Protection in 2023, China (SKLGP2023K028); The Key Research Base of Social Sciences in Sichuan Universities--Research Center of Science & Technology Innovation and New Economy in Chengdu-Chongqing Dual-city Economic Circle (CYCX2022XSXRYB01).

## Conflict of interest

The authors declare that the research was conducted in the absence of any commercial or financial relationships that could be construed as a potential conflict of interest.

## Publisher’s note

All claims expressed in this article are solely those of the authors and do not necessarily represent those of their affiliated organizations, or those of the publisher, the editors and the reviewers. Any product that may be evaluated in this article, or claim that may be made by its manufacturer, is not guaranteed or endorsed by the publisher.
